# Physical Activity Participation in Rural Areas: A Case Study

**DOI:** 10.3390/ijerph19031161

**Published:** 2022-01-20

**Authors:** Celia Marcen, Eduardo Piedrafita, Rubén Oliván, Irela Arbones

**Affiliations:** 1 Faculty of Health Sciences, Universidad San Jorge, Campus Universitario de Villanueva de Gállego, Villanueva de Gállego, 50830 Zaragoza, Spain; rolivanpueyo@usj.es; 2 National Institute of Physical Education of Catalonia (INEFC), University of Lleida, 25192 Lleida, Spain; marbones@gencat.cat

**Keywords:** environment, outdoor sports, healthy lifestyles, semi-rural, informal sport

## Abstract

Sport participation and physical activity promotion have been intensively studied, especially regarding large cities. However, in many cases, rural municipalities have comparatively reduced services, including health care, due to the low profitability that these have for companies and entities. In this sense, the purpose of this article was to describe a case study of the promotion of physical activity in rural areas based on the results of a European project. Carried out in a rural municipality of around 8500 inhabitants with a population density of less than 25 inhabitants/km^2^, the project’s purpose was the promotion of autonomous physical activity among its inhabitants. For this, a diagnostic analysis of the sports areas of the environment and a survey of physical activity habits among the population were carried out. A series of routes were designed, marked, and signposted, and canopies with explanatory posters about the possibilities of healthy physical activity and recommendations were added. Free-use facilities were installed, and opening events were carried out in such a way as to stimulate sports practice among the population. This article presents the results obtained from the analysis, as well as the possibilities of replication in other municipalities with similar needs.

## 1. Introduction

Inequalities in general, and in health services and opportunities between urban and rural areas in particular, have been thoroughly studied [1,2]. Urbanization and migration have been highlighted as the most relevant aspects in recent developments regarding social inequalities in health. Though many cities have problems due to overcrowding and the unequal distribution of resources, the rural world is lacking in terms of access to quality services, facilities, and programs. A study conducted in Canada found that even though children, adults, and seniors in rural areas spent more time doing outdoor activities than those who lived in cities, the researchers identified higher mortality rates, decreased life expectancy, a greater incidence and prevalence of morbidity, and poorer self-reported health status in rural populations [3].

In many European countries, the levels of depopulation in certain regions have become serious and urgent problems for which solutions are not simple. In the case of Spain, this phenomenon has been called “empty Spain” and entails both health and well-being issues for its inhabitants, especially women [4].

Rurality can be conceptualized in terms of size and/or density. The Spanish Law of Sustainable Development of the Rural Environment (approved on 13 December 2007, see reference) defines the rural environment as the geographic space formed by the aggregation of municipalities or smaller local entities with a population of <30,000 inhabitants and a density of <100 inhabitants per km^2^, which is similar to parameters of the Eurostat or the Organization for Economic Co-operation and Development (OECD) [5].

When talking about health, well-being, and quality of life, most authors point to physical activity and sport, among others, as means to both maintain health and prevent future illnesses [6,7].

Participation in physical activities has maintained a series of structural inequalities throughout Europe; experts have highlighted decreases of physical activity with age, a gendered distribution (men participate in more sport in all age ranges, especially in team and strength sports; women prefer gymnastics and swimming), and a positive relation between socioeconomic and educational levels and sport participation. In this sense, women have a greater risk of low private participation due to the unequal distribution of household and care responsibilities, as well as scarce sports socialization in the childhood of the oldest [3,4].

Additionally, sport is an urban phenomenon, with the greatest use of rural areas being by city dwellers who go out to practice outdoor activities in nature [8,9,10]. European and national sport participation surveys have shown that the practice of outdoor activities in non-competitive, informal groups with aims for the improvement or maintenance of health are growing in all of the countries of the European Union [8]. Public spaces such as streets, parks, and mountains are favored locations to play sports in most countries.

Eurostat data for 2021 showed that participating in any cultural or sport activity is quite popular within the European population (EU-27); 66.2% of its citizens played a sport in the last 12 months [11]. However, when considering the degree of urbanization, this percentage was found to be slightly reduced in rural areas in Europe (60.1%) and drastically reduced in Spain (50.7%). However, recent research highlighted social class as the main barrier to playing sport, with one’s place of residence presenting little influence [12].

Spain is the EU-27 country with the second-highest percentage of the population that says they do not engage in physical activity due to economic problems (the highest percentage is found in Greece). In “empty Spain”, the lack of services and infrastructures in general (the ‘desertification of services’), as well as for sport facilities and programs, accentuates these deficiencies [13].

Although different surveys on sports participation in Spain have been used to analyze the obtained data according to age, gender, employment, or educational level, among others, no analysis that considers the urban/rural factor as an explanatory of certain sports and health habits has been carried out at the national level. The 2020 survey was also greatly influenced by the global pandemic situation [9,10].

The data from the 2020 survey can be downloaded by province capital, non-province capital municipalities with more than 50,000 inhabitants, and municipalities with 50,000 or less inhabitants. However, there are no data for municipalities with smaller populations. Sport participation in municipalities with under 50,000 inhabitants has been reported to be close to the Spanish average. People living in these towns reported playing sport in a higher percentage than the average, but these data were conditioned by COVID-19, as medium and large cities had more mobility limitations and less access to the outdoors in close environments. Taking the 2015 data as a reference, sports participation in Aragon was found to be three percentage points below the national average.

In Spain, some studies on rural sport have been carried out, especially from the perspective of sport for development [14,15]. In them, it has been pointed out that the rural environment is often seen as a factor of local development through sports tourism, generating infrastructures more focused on visitors than inhabitants. In other words, rural sports are seen as a means for economic development rather than as a tool to improve the local quality of life. As such, the authors recommend the design of public policies in which all stakeholders intervene and consider all perspectives of sport for development (related to not only the economy but also to the health and physical, psychological, and social well-being of its inhabitants).

The Report on Inequality in Aragon stated “that inequality (socio-economic and demographic) between urban and rural areas of our region will grow” and “specifically, access to basic services will be one of the most important elements to stop and try to reverse depopulation in some areas of our region” [16] (p. 66).

Accordingly, the purpose of this study was to use a case study to analyze the sports participation of a Spanish rural municipality, based on the actions of a European project to promote physical activity in rural areas. Given the lack of scientific studies in small and medium size towns, this research provides new knowledge and collaborates in the establishment of evidence-based public sports policies.

The A-TWIN project (Active Twinning for Enhancing Physical Activity in Rural Areas- Erasmus+ Sport Ref. 613158-EPP-1-2019-1-ES-SPO-SSCP) promotes physical activity and active lifestyles in rural environments, connecting local culture, environments, and sports through a diagnosis studio, a scientific analysis of the sport habits of the population, and a series of interventions. This project is based on three pillars: (a) outdoor physical activities and sport for everybody; (b) cultural learning based on common interests, history, and ties between twinned cities; and (c) environmental education through outdoor activities in a rural environment [17].

The objectives of this research were:

1. To understand the specific needs of a rural population regarding physical-sports activity practice o understand the specific needs of a rural population regarding physical-sports activity practice.
2. To plan, implement, and evaluate a series of actions related to the interests of a rural population and recommendations for healthy habits.
3. To contribute to the establishment of public policies based on evidence related to healthy physical activity in rural populations of under 10,000 inhabitants.

## 2. Materials and Methods

The case-study methodology is a process-based, systematic, and in-depth investigation to study the “real-life” of a phenomenon closely linked to its environment [18]. This approach is especially beneficial when evidence-based polices are questioned [19]. This study was based on the inductive approach of Stake [20], which seeks to find patterns to create theories while recognizing the role that participants have in the co-construction of these theories.

The selection of our cases followed the logic of theoretical sampling [21], with the intention to introduce small rural areas the sport participation and physical activity promotion in rural areas. The case studies also included interventions at a local level and the sharing of best practices at the international level. The studies targeted the outdoor physical activities sector and were located in three municipalities connected by a cultural agreement [17].

**Figure 1:**
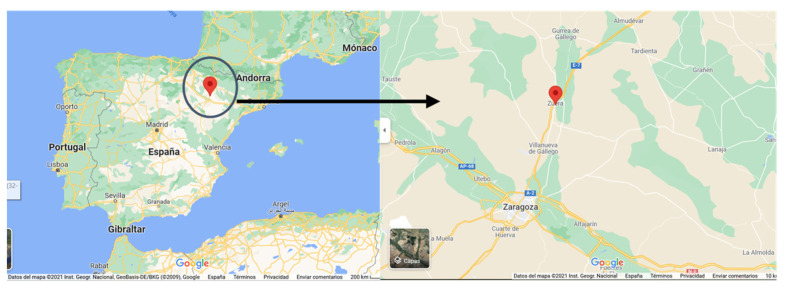
Zuera municipality location (from Google Maps©; data from ©2021 Inst. Geografía Nacional; accessed on 27 November 2021).

The A-TWIN project aimed to promote physical activity in rural areas by connecting physical activity, culture, and environment. The results shown here correspond to a single municipality, Zuera, in the northern Spanish region of Aragon (Figure 1).

Zuera can be considered rural due to its population (8565 in 2020) and density (25 inhabitants/km^2^). Though agriculture and farming continue to have great economic weight (52.62% of the total area of the municipality corresponds to Utilized Agrarian Surface—SAU), Zuera’s location near the province capital (Zaragoza) and the existence of industrial parks have led the Zufarian population to primarily work in industries and services related to the foreign population which account for around 23% of residents [22].

Our methods were intended to generate a holistic understanding of physical activity participation in a rural town. The data we collected comprised the results of a diagnosis study with the local government responsible for the sports, culture, and environment areas (*n* = 3); a sports participation survey (*n* = 412; valid *n* = 393); and an outdoor activities (workshops) program (*n* = 131; valid = 129).

### 2.1. Stage 1: The Diagnosis Studio

The first step of this research was conducting a diagnosis studio with the city council. Sports, culture, and environmental areas were contacted to answer a questionnaire about municipality resources (the Diagnosis Questionnaire can be seen in Appendix A). The dimensions and topics to discuss were:

(1) Sport facilities and spaces for physical activities.
(2) Sport habits.
(3) Offered services in outdoors.
(4) General topics.

The unit head from the three areas were contacted by phone by the research team to inform them of the project objectives and the need for their support (previously, the town major accepted the municipality participation in the A-TWIN project as partner and informed of the three areas in which they were going to be contacted). The questionnaire was sent to them by mail. After receiving and analyzing the information, an online video conference was arranged to discuss the data, resulting in a SWOT analysis as a starting point for the survey of sports habits (stage 2).

### 2.2. Stage 2: Sport Participation Survey

Following the SWOT analysis, a sport participation survey, with special focus on outdoor physical activities, was conducted. Researchers carefully analyzed existing sport participation surveys, especially national and local questionnaires [12,23]. In addition, the research team conducted an analysis of the municipal website from which the offered activities were extracted, as well as the information on the practice spaces collected in the first questionnaire of the A-TWIN project needs analysis [24].

Based on this extensive research, a first version of the questionnaire was designed following the methodological orientations of Díaz de Rada; expert validation (in which six people representing different genders, age ranges, educational levels, and types of employment participated) was then carried out [25,26,27].

The validation consisted of two steps. The first one only aimed at measuring time based on the two response itineraries raised by the questionnaire (whether the subject performed physical activity or sports). Next, the experts were asked to repeat the operation, carefully reviewing the questions, answers, structure, sequencing, etc., in order to analyze relevance and adequacy. Based on this analysis, the experts were asked to complete the following table, which indicates everything that could contribute to the improvement of the instrument (Table 1).

As a result of the collected information, a total of seven modifications were made to the questionnaire, with five suggestions being rejected for different reasons (technical, scope, means, etc.). Then, the final version of the questionnaire was delivered [27]. A pilot test using the final instrument was carried out with a diverse group of eight people.

The questionnaire was distributed to all associations (cultural, sports, and social) appearing in the municipality census, along with an explanation of the project and a letter of invitation to disseminate it among associates. In addition, the Zuera city council publicized it through its municipal website and social networks so that anyone who met the conditions could respond if they desired.

The questionnaire was opened for one month (from 12 November 2020 to 11 December 2020). The first screen showed the information, explanations of the project and the questionnaire, the use of data, and a guarantee of anonymity and privacy, and it was required that participants read and accepted the terms to continue.

The data we collected included attitudes towards sports practice and participation and the perceived relationship between physical activity and health. Due to the COVID-19 pandemic, the survey could not be conducted face to face, and we decided to use an online survey using Google Forms.

The resulting database consisted of 393 observations belonging to different individuals who had been given a survey with 18 questions. From these questions, many of which were multiple-response, a database with 72 variables was created. Of the 72 collected variables, 27 were ordinal factors (Likert scales or similar), 44 were non-ordinal factors, and 1 was free text. The objective of the analysis was to contrast (i) the existence of associations between the different collected variables, and (ii) the existence of the possible groupings or clusters of individuals based on their sporting habits [28].

After processing the database to adapt it to a format and coding that could be treated with statistical software, a general description of the data, and a specific description of the Likert-type variables corresponding to questions 5 (sport meanings) and 12 (sport values) of the survey were written. To study the associations of the different variables with each other, an analysis was carried out using Goodman and Kruskal tau. The results are presented as a heat map and a numerical matrix. To study the possible grouping of the different individuals, a specific grouping algorithm was used for categorical variables using the dissimilarity matrix based on Gower’s distances. All analyses were carried out using R software (version 4.1.1, R Foundation, Vienna, Austria) and the clickR (version 0.8.0), Likert (version 1.3.5), and cluster (version 2.1.2) packages.

### 2.3. Stage 3: Outdoor Activities Program Design

Designing the workshops for the program to be carried out in the natural environment required exhaustive planning and preparation in order to meet the objectives and encourage the motivation of the participants [29].

When choosing the project activities, adherence to physical activity and the sport participation survey results were analyzed (stage 2), reflecting the trends of outdoors in informal and non-competitive groups that use public spaces [30]. For these reasons, orienteering, Mountain Bike (MTB), Nordic walking, trail running, and climbing (individual activities that can be carried out in groups) were selected for encourage physical activity in the population of the rural municipality under study.

Based on the topographic information of the area and a previous analysis of the existing routes, as well as an analysis of the context of the study municipality, different routes with different degrees of difficulty and lengths were designed to be traversed by foot and bicycle. These routes were signposted using pictograms that provide information on the characteristics of the routes and promote good practices, using sustainable materials from nature to reduce environmental impacts [31].

The signposting and promotion of trails increase their use [32]. As such, to promote the different routes, some days were organized to enable the discovery of the routes.

The participants were recruited with the use of posters in the main areas of the municipality, as well as the use of the social networks of the project, the council itself, and citizens (since being enrolled in an activity implies a greater degree of social commitment and has effects on the continuity of said practice) [33]. Specifically in young people, directed sports practices promote social relationships and help to consolidate sports habits in adulthood [34]. However, motivation and satisfaction are driven by the ability to self-manage activities [30]. The proposed activities were as follows.

- To promote the walking route, a Nordic walking activity (a modality that uses the use of poles in order to recruit the muscles of the upper body and thus increase caloric expenditure) was carried out [35]. The session was conducted by an instructor specialized in Nordic walking ince the support of instructors and group colleagues helps to improve self-esteem and benefits well-being and at the end of the activity with satisfactory sensations [36].
- Orientation races are a progressively growing physical-sporting activity due to their competitive, recreational, and educational nature, as well as their ability be carried out in educational, rural, and urban areas [37]. In this case, the designed route was marked through QR codes (“Quick Response Code”). These codes, which allow for different actions such as opening a website, reading a text, or opening an image or pdf, were made to contain questions related to the flora, fauna, and cultural aspects of the practice area. To promote the orientation circuit, different open days were planned with local primary and secondary schools since this type of initiative, in which physical activity and the use of information and communication technologies are mixed, leads to greater interest and motivation in participants [37,38].
- At the same time, following the same methodology, a trail running day combined with suspension strength training was carried out. The combination of strength work with aerobic work, a method known as concurrent training, reduces the risk of musculoskeletal injuries [39], optimizes sports performance [40], and improves health and quality-of-life parameters at all ages [41,42,43]. In both older men and women adults who experience loss of muscle mass, the risk of falls increases, and balance decreases with age, even in those who have been advised to aerobically exercise several days a week. As a result, it has been proven that concurrent training has a place in health improvement programs [41,42]. Similarly, this type of training helps to reduce the percentage of body fat mass and central adiposity, thus improving the anthropometric profiles of young people with obesity [43,44]. Its practice is also recommended in prepubertal boys and girls [45,46].
- The promotion of sport participation was also carried out through a climbing activity stimulated with displacement games at a small urban climbing wall that had been installed in the town. Rock climbing/bouldering is an increasingly popular sport among young people and one of the recommended activities for adults in national health promotion programs [47]. Its non-competitive practice helps fight diseases such as obesity, and its moderate or vigorous physical activity allows for the improvement of both the anaerobic and aerobic fitness of the practitioner since the intensity of the exercise is comparable to that recommended by the American College of Sports Medicine to maintain good cardiorespiratory fitness and to increase both muscle strength and flexibility [48,49].
- The same marked routes for Nordic walking and trail running were used for an MTB session, with a lesson on how to use a repair station and how to take advantage of the municipality’s surroundings to enjoy different levels of difficulty with bicycles.

Signposting and poster examples from the routes and activities can be seen in Appendix B At the end of each of the activities led by specialized personnel, the participants completed an evaluation sheet that was designed based on the objectives of the European project (Evaluation sheet could be found in Supplementary File S1). A total of 131 participants carried out the different activities, and the authors obtained 129 valid evaluations (98.5%).

## 3. Results

### 3.1. Results from the Diagnosis Study (Stage 1)

**Figure 2:**
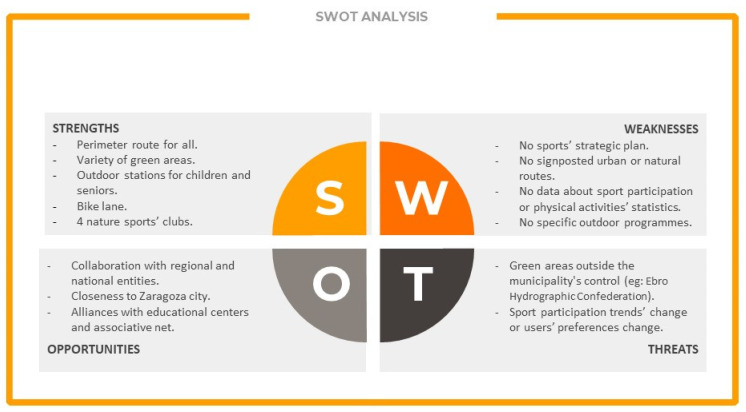
SWOT analysis of physical activity resources in the Zuera municipality.

Once the questionnaires were filled in and the results were analyzed by the research team, they were discussed with the town council representatives and a SWOT (strengths, weaknesses, opportunities, and threats) analysis was developed to reflect the results of these processes (Figure 2 presents the results of this study. A longer list was considered, but it reported on aspects of comparison between the partner municipalities of the A-TWIN project so was of no interest for this study).

Internal positives (strengths) were reported to be related to the existence of a perimetral route suitable for all, the variety of green areas in the municipality (parks, the riverbank, forest …), and the existence of four sports clubs related to activities in nature. Internal negatives (weaknesses) were reported to be related to the lack of a sports’ master plan in the municipality, signposted routes, data about sport participation, and (despite the fact that the municipality offers a considerable variety of physical activities), and nature-based studies (they had mainly been oriented to fitness). At this project level, only the internal factors were considered since the external factors were less controllable by the research team and subject less to intervention from sports public policies.

### 3.2. Results from the Sport Participation Survey (Stage 2)

Those who answered the survey comprised 54% women, 45.8% men, and 0.2% ‘others’. The age group that answered the most was participants aged 25 to 44 years old (46.8%), followed by participants aged 45 to 64 years old (29%), and young people between 14 and 24 years old (24.2%). Most of them had lived in Zuera for at least 10 years (only 12% of those who responded had lived in the municipality for less time). It was reported that 71% were active workers, and most lived as couples with children (36.7%) or their parents (31%); to a lesser extent, respondents live as couples without children (17%). A general descriptive analysis of the data is presented in Appendix C.

General interest in sport among the surveyed population was high (36.1%) or very high (27.5%). Despite this, almost a third (29%) of the participants considered sport to be of little or no interest to them.

**Figure 3:**
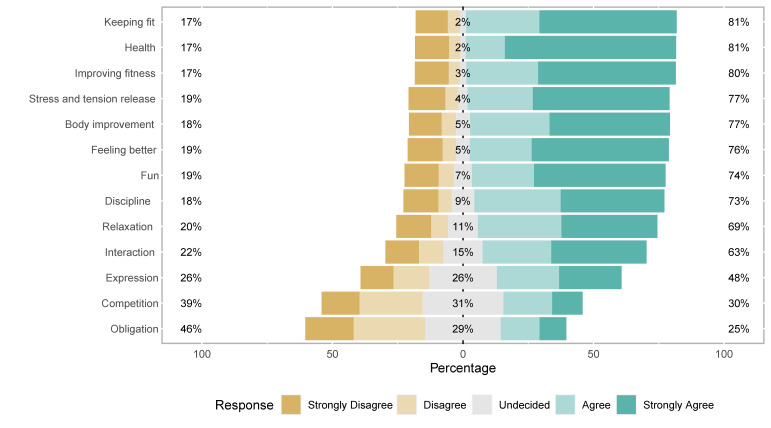
Sport meaning’ identification (answering ‘Sport for you is…’) in percentages.

In relation to the meanings of sport, with those who said they strongly agree used as a reference, the strongest identification was found to be between sport and health (65.5%), followed by fun, keeping fit, improving physical fitness, getting rid of tension and stress, and feeling better about yourself (all around 50%). Figure 3 shows the identification of different meanings of sport, revealing that the definitions of sport as an obligation and competition were the least accepted.

We found that 85% of those who responded practiced physical activity or sport. The percentage of men athletes was slightly higher than that of women, with a gender gap of −6%. This difference can be considered moderate compared to the national average (11.6 percentage points difference) or the nearest city (12.7 percentage points). Regarding lifespan, young women were those who reported practicing less physical activity in comparison to men (42.3% vs. 57.7%, respectively, for participants between 14 and 14 years of age). This relationship was found to be reversed as age advanced, with a difference of only 1% between men and women in the age group of 25 to 44 years of age. Among those over 44 years of age, women practiced sports to a greater extent than men (64.4% practiced sports), mainly due to the roots that walking has among the population of women in the villages.

Young adults between 25 and 44 years old reported practicing sports the most, slightly decreasing with advancing age and young people between 14 and 24 years old reported practicing sports to a lesser extent; there was an age gap of more than 10 percentage points.

Among those who do not practice, laziness (34.5%) and lack of time (29.3%) were the most reported reasons, while current offer was reported to a much lesser extent (12%). It was reported that 8% of those who did practice sports do not like them, while health reasons or COVID-19 only represented around 5% of the responses. A lack of company and financial means were minimally reported.

Two-thirds of those surveyed said that they would like to engage in more physical activity than they currently do. Almost half (48.5%) considered their state of health as good, while 25.4% considered adequate and 17.1% stated that it was very good. It was found that 9% considered their state of health poor or very poor. When asked about their fitness, a quarter perceived it as poor or very poor, 32.5% perceived it adequate, and 44% perceived it as good or very good.

The most practiced physical activities were found to be walking (61%), hiking (39.7%), running and cycling (31.3%), swimming (24.7%), bodybuilding and fitness sessions (23.5%), skiing (17%), and paddle tennis (15.3%). The preferred place to practice was nature (29.9%), followed by municipal sports facilities (23.7%), and (to a lesser extent) private facilities (7.6%). In addition, it was preferred to practice in a group (20.8%) in comparison to practicing alone (13.2%), although 10.3% reported playing sports both alone and in a group.

Slightly more than half of those who practice sports reported doing so in the municipality itself (51.6%), and men were found to carry out activities in it to a greater extent than women.

**Figure 4:**
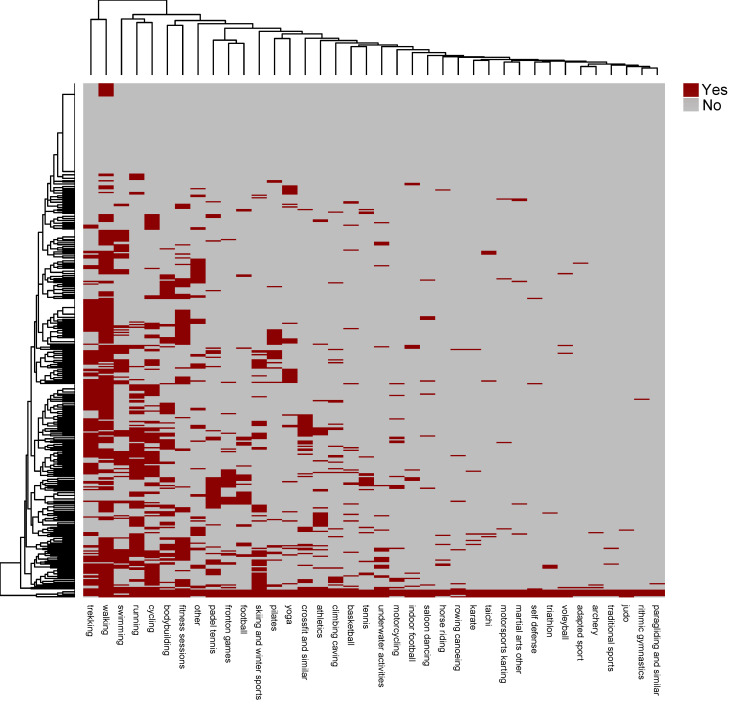
Heatmap of different responses to questions about sport participation (only for those who answered that they play sports).

For descriptive purposes, the answers to question 8 (‘what sport(s) do you practice?’) are represented in a heat map, that is, a colored matrix based on the intensity with which the different variables are presented. In this case, the sport modality practiced. (Figure 4).

The heat map orders, from left to right, the most practiced physical activities; the top was reported to be walking for fitness (61%), followed by trekking (39.7%), running (31.3%), cycling (31.3%), swimming (24.7%), and fitness and bodybuilding (23%). Skiing and winter sports stood out (17%), as did the booming sport of paddle tennis (15.3%). Around 10% of participation was reported for yoga, Pilates, athletics, football, or fronton games.

Regarding the habits and periodicities of practice, 54.8% participated in sports a minimum of three days a week, and less than 4% reported performing physical activity sporadically (less than one day a week). In addition, most practiced throughout the year (71.9), although a not inconsiderable 18.6% only exercised during the week. Those who reported playing sports only on weekends comprised 7.8%, while those who reported only playing sports on vacation accounted for less than 2% of the responses.

Consistent with the low identification with competition, only 35.3% of those who practiced sports competed, and only 16.8% of these did so in federated competitions. The rest stated that they competed in a popular way or with family or friends.

**Figure 5:**
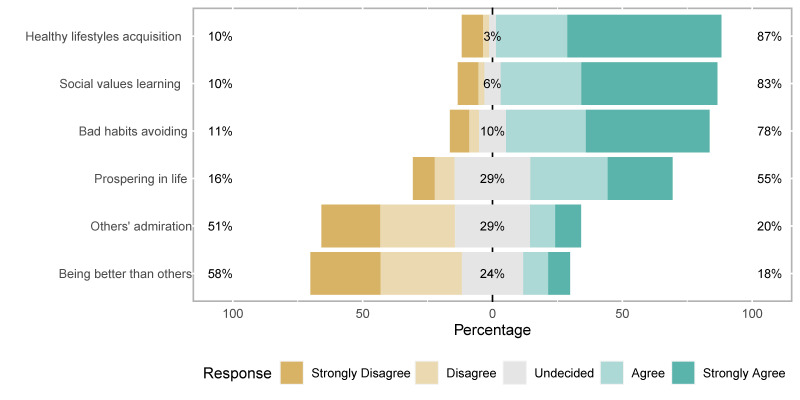
Sport values (answering ‘what can sport do for the youth?’).

When asked about the values that sport provides youth, the acquisition of healthy habits was supported by the majority (59.3% completely agree and 27.5% agree). The perceived sport values can be seen in Figure 5.

**Figure 6:**
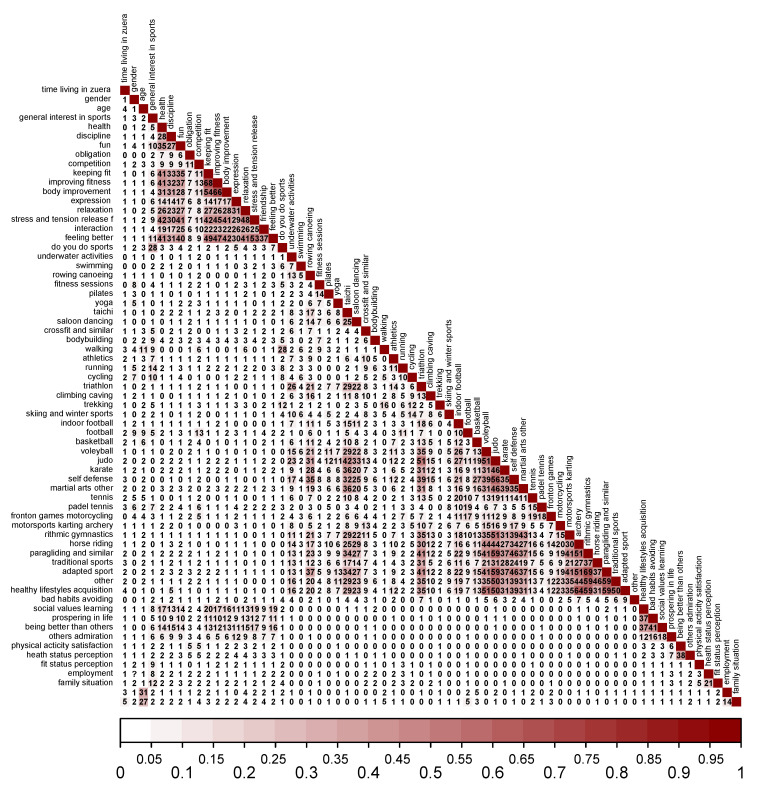
Matrix with the results of the Goodman and Kruskal tau statistic. The highest association values are marked with a darker red color (values of tau are expressed as percentages in the figure).

Given that all of the variables in the survey were categorical, with some ordinal and others nominal, the Goodman and Kruskal tau statistic was used to study the possible associations between them. Given the enormous number of available variables (which implied the calculation of 2211 measures of association between all pairs of variables), the results were organized in a matrix with the values of the tau statistic and are represented in a graph (Figure 6). The red gradation indicates the level of association between variables (dark red corresponding to the maximum value, close to 1, and white to the minimum, close to 0) in a visual way.

In the results of the analysis, it was observed that, in addition to some associations between the practices of some types of sport, there were also associations in the answers to the question regarding perception of sport and the question regarding perception of sport for youth. Additionally, there were logical associations between work, family status, and age; between fitness and health; between playing sports and interest in sports; and between playing soccer and thinking that sport is competition.

Of special interest to this research is that no association appeared between gender and other variables such as meanings and values of sport, modality practiced, or general interest in sport (τ ≤ 0.09), and there was only a low association with walking and age (τ ≤ 0.11). There was a low association between general interest in sport and some meanings given to sport such as ‘fun’, ‘feeling better’ (τ = 0.10 and τ = 0.11, respectively), and there was a moderate association with sports participation (τ = 0.28). Additional moderate associations between the different meanings of sport could be observed, with especially high associations between staying in shape and improving physical form, (τ = 0.68) and between improving the body and maintaining physical form (τ = 0.66). Feeling better about yourself through physical activity was associated with all meanings of sport (τ of between 0.31 and 0.53), except for ‘obligation’ (τ = 0.08) and ‘competition’ (τ = 0.09). Similar to the meanings of sport, the different recognized sport values were lowly (‘prospering in life’ with τ between 0.06 and 0.18) or moderately associated with one another (the relation between the acquisition of good habits, avoiding bad habits, and social value learning ranged from τ ≤ 0.37 to τ ≤ 0.41). However, there was no association between the meanings and values of sport and the modality practiced (τ ≤ 0.06), apart from a slight association between ‘practice of football’ and ‘competition’ (τ = 0.13). Finally, ‘being better than others’ presented a moderate association with ‘other’s admiration’ (τ ≤ 0.38).

The hierarchical clustering analysis performed using Gower’s distance-based dissimilarity matrix showed four different groups of individuals who participated in the survey.

- Group 1—Red: This group was composed of 65 individuals, most of them not practicing any physical activity or sport, who strongly disagreed with most of the proposed meanings and values of sport. They showed a negative perception of sport.
- Group 2—Green: This group was the smallest, comprising 6 individuals who said that they frequently or very frequently practiced all sports and were in good health and fitness. The veracity of their answers has been called into question.
- Group 3—Blue: This group represents the sporty people (156 individuals) with high frequencies of weekly activity. However, in this case, the number of practiced sports was smaller than that of group 2.
- Group 4—Turquoise: Group 1 was primarily of people who do not play sports (166 individuals). The difference between groups 1 and 4 is their identification with sport meanings and values (as shown in Figure 3 and Figure 5, respectively), with group 4 individuals reporting positive perceptions of sport even though they do not practice sport.

**Figure 7:**
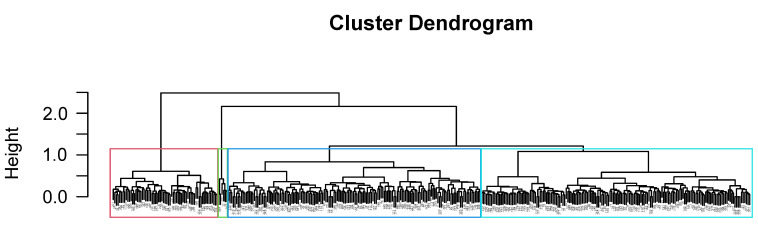
Dendrogram with the clustering results of the different individuals based on their responses.

Presented below is a dendrogram graph with the results of the hierarchical clustering analysis (Figure 7).

### 3.3. Results from the Activities’ Program (Stage 3)

It was found that 131 people participated in the different workshops, and they were evaluated in relation to the objectives of the project. Table 2 shows the descriptive statistics of the participants.

The results of the evaluation of the activities showed that a large majority linked them with education (85.3%), 13.2% needed more information to be able to assess this item, 1.6% did not answer, and there was no negative answer in this regard. Women evaluated activities as educational to a greater extent than men (+6%).

By age group, young people between 14 and 24 years old considered the activity as educational to the greatest extent (96.2%), followed by adults between 25 and 44 years old (89.5%), and adults of 45 to 64 years old (86.7%). In contrast, young people under 14 years of age reported the least support for this statement (79.7%).

Regarding the way of carrying out the activity, the majority of participants carried it out with company (82.9%), though a percentage worth considering began in a group and then continued at his/her own pace and/or level as the activity developed (10.9%). We found that 3.1% started alone and then joined another person or group. Accompanied practice preference was similar for both men and women. However, considerable differences were found by age group; those under 14 years of age comprised the highest percentage (91.3%), followed by adults from 25 to 44 (84.2%), young people from 14 to 24 years (76.9%), and finally the oldest group (53.3%). In this regard, it should be noted that children under 14 years of age could only participate in orientation, as a school activity that was carried out in pairs.

Regarding the repetition of the activity while modifying some aspects, only 3.1% did not want to repeat it, 28.7% wanted to repeat it in a similar way in a different environment, 24.8% wanted to repeat in a different environment with different characteristics, and 24% wanted to take advantage of the spaces and routes created to participate in new activities.

By gender, men reported wanting to carry out the same activity in a different environment with the same characteristics to a greater extent (33.8%) than women, who preferred to repeat the same activity in the same environment (27.9%). By age group, those under 14 preferred to repeat the activity in another similar environment (30.4%), young people from 14 to 24 years old wanted to take advantage of the experience and propose new activities (46.2%), and adults wanted to repeat the activities in different environments with different characteristics (36.8% in the 25 to 44 age group and 53.3% in the 45 to 64 age group). Only the youngest (under 14 years), especially the boys (5.8%), reported any desire to not carry out activities of this type again.

The level of difficulty was optimal for the majority (60.5%), while 23.3% would have liked more difficulty, 8.5% would have liked lower difficulty, and 7.8% did not answer the question.

While 30.9% of men asked for a higher difficulty level, only 14.8% of the women reported being more comfortable with the offered level (68.9% considered the difficulty adequate).

By age group, adults were the ones who most asked for a higher level of difficulty (47.4% in the 25–44 age group and 40% in the 45–64 age group). Those between 14 and 24 years old considered the level to be adequate to the greatest extent, while, there was a greater distribution of responses among those under 14 years of age (56.5% considered it adequate, 18.8% wanted to increase it, 14.5% wanted to lower it, and 19.1% did not answer).

The Likert scale of faces (from total dislike to total approval) showed the greatest support for the ideas that the activity improved health (63.6% fully agree) and that it maintained or improved one’s physical shape and was fun (60%). To a lesser extent, respondents thought that the activity made them feel good (45%), and even fewer tought that they could learn about culture through it and other related sports activities. The greatest disapproval was reported for the ability of these activities for relaxation or to relieve tension and stress (around 9%).

It was found that 48.8% of the participants believed that these activities linked physical activity and the environment, and this relationship seemed positive to them; 58% totally agreed that they would like more activities of this type to take place in their municipality. Combing those who strongly or totally agree results in a percentage of 79.8%.

## 4. Discussion

This study demonstrates the potential for rural municipalities to enhance physical activity, promote healthy lifestyles, and reduce premature physical activity participation abandonment in youth. Solid theoretical and evidence-based bases are not frequent in rural areas, which are different than urban areas in many ways, including the statistics that are offered for the design of public policies in the field of health. In addition, the possibility of linking these activities to knowledge and awareness of the environment and local culture are a great strength of this project.

The level of sports participation in Zuera was found to be unusually high (85%) compared to the averages for the region (50.5%) and the national (53.5%), national rural areas (50.7%), Europe (66.2%), and rural European areas (60%) levels [10,11,12,13,22].

In the same way that there is an increasing identification of sport with health at a global level (with talk of a “healthization” of society in general [45,50]), Zufarians were found to identify sport as healthy to a high degree, as well as with obtaining or maintaining physical fitness. Previous studies have indicated how these factors have displaced meanings and enjoyment motives in different contexts [48,51,52], but the number of participants who strongly agreed that sport is fun was somewhat higher than expected.

The trends that have appeared in the national and European levels of the practice of physical activity in public and outdoors places with non-competitive and informal groups were maintained in the studied population, sometimes to a greater degree, probably due to the ease of environment use [53].

Sport as an obligation was found to have a low acceptance (25%), as in other surveys that have found that this percentage has been growing in the last five years. The main barriers to practice are a lack of motivation and work–life balance (lack of time), similar results to those obtained in the nearby urban environment and other European regions [11,13,22]. Current offerings or economic problems do not appear to be a limiting factor.

With regard to the cluster analysis, four distinct groups clearly appeared: that of “potential athletes” (Cluster 4—Turquoise) who do not play sports but perceive it in a positive way, the “sporty” (Cluster 3—Blue), who perform various sports, the “negative non-athletes” (Cluster 1—Red), who not only do not practice sports but also do not value them positively at all, and the “total athletes” who say they practice all of the sports on the list and are in an excellent state of both health and fitness (Cluster 2—Green).

It seems unrealistic to think that there were six individuals who practice every one of the listed sports, so these results may have been caused by errors when selecting the boxes or intentional attempts to contaminate the sample. Therefore, this group can be eliminated, and it is proposed to design programs and public policies for the other three groups with different interests, evaluations, and motivations for physical activity: (1) “potential athletes”, for which we could offer activities and guidance until they are engaged and autonomous, as well as promoting balance between work, care responsibilities, and their leisure time (including physical activity); (2) the “sporty”, for which we could improve the variety and level of activities, creating new routes or promoting new areas around the municipality; and “negative non-athletes”, for which the approach should be aimed at a cognitive level, increasing their knowledge about the benefits of sports combined with positive experiences related to physical activity. Special attention should be given to young people, since they were found to practice sports in a much lower percentage than expected (in comparison with previous studies, they reached 8 percentage points below the mean).

The proposed activities logically had better evaluations when they were voluntarily completed in comparison to the respondents under 14 years of age who engaged with them as a school activity.

In all cases, the results showed the aforementioned links between sport and health and between sport and physical fitness, which also appeared in the survey. Regarding the final question about interest in similar activities (while considering the results of the evaluation in terms of environment and difficulty), there was a wide demand for this type of guided activity that could later lead to autonomous practice with greater knowledge.

The results confirm the hypothesis that when applied in cities or in segmented samples at the regional or national levels, surveys are not useful in the understanding and analysis of sports habits or the proposal of effective strategies in small municipalities, which have their own dynamics [53].

Additionally, these results reflect the need to install infrastructure, spread information (something that has been managed through information posters), and implement training [54]. As such, a future development direction could include the training of trainers and the periodic revitalization of different spaces and facilities.

In contrast with the previous literature, a relationship between physical activity in nature and mental and emotional well-being was not found, as both the survey and evaluation of the activities showed these to be less valued aspects than the fact that sport enables health and the maintenance of the physical form [55].

### Limitations

Caution must be exercised when considering these data due to the limitations discussed in the methodology section (such as the change from the face-to-face survey to the online survey due to the pandemic, which may have resulted in only the people most interested and involved in sports to agreeing to participate). This problem was neutralized once we analyzed the data of those who actually did play sports, as has been carried out in past research.

Regarding the cluster analysis, we did not have enough data (due to the limitations of the methodology) to assess whether the individuals belonging to cluster 2 genuinely possessed their opinion or were exhibiting ‘troll’ behavior.

## 5. Conclusions

This project was designed by linking actions so that the analysis of needs led us to design the Zufarian population sport participation survey, analyze the results, and devise the activities program. Finally, our evaluation was linked to the project objectives, thereby closing the circle.

1. In relation to the objectives of this work, this project has shown how the needs and demands for physical-sports activities in rural areas are specific and that there are differences between the trends observed in different studies at the national and European levels (where urban populations weigh heavily in the composition of samples). Therefore, there is a need to deepen the knowledge of these needs as a way to improve the quality of life of inhabitants. The actions proposed in the program were adequate, valued by the participants, and useful when planning specific public policies in the area of healthy habits at the municipal level.
2. The methodological limitations caused by COVID-19, such as needing to carry out the survey through an online questionnaire, resulted in the percentage of people who perform physical activity appearing unrealistic (85%), probably due to the fact that the respondents who were more motivated and involved in the subject answered to a greater extent than their counterparts. In this way, some of the results cannot be generalized. However, the methodology applied in the design of the project, the previous needs analysis involving the main stakeholders, and the evaluation of the implemented activities may allow these results to be extrapolated to other rural municipalities and be useful in the design of public policies at the local level.
3. The main strengths of this study are its results derived following the evidence-based designed of complete program, which highlighted the identification of sport with health and enjoyment among the study population and the role of physical activity as a means to acquire and maintain healthy habits. These results were obtained in a convergent way via both surveys and in the evaluation of the proposed activities. Regarding physical activity preferences, in agreement with global trends, the results pointed towards a taste for outdoor activities that are individual (walking, running, cycling, etc.) but can be carried out in groups. The success in designing the activities is reflected in the positive evaluation of them in relation to the objectives. Of the 131 participants in any of the five proposed activities, there were no negative responses to the link between sport and education, the intensity was valued as adequate by the majority, and only 3.1% of the participants did not wish to repeat the same activity or like the one performed.
4. A large percentage of those who practice sports reported doing so in the municipality itself, so initiatives such as the one presented here can be useful to provide better service and to attract those who currently prefer to play sports outside. By offering more and better services in the nearby environment, the quality of life of the inhabitants is favored, movements are limited, and a greater knowledge of the environment and sense of belonging is promoted.

Sport can contribute to these improvements largely due to the values that the survey population attributed to them in the establishment of healthy lifestyles, the avoidance of bad habits, and the learning of positive social norms and values. Finally, with regard to the orientation of public policies, the obtained results point towards different strategies that consider the following.

- The main barriers of laziness and the lack of time; for the former, campaigns should be established at a cognitive level (on the benefits and fun of engaging on physical activity, the ease of participating in the specific environment, etc.) while considering the Prochaska and DiClemente change model [56]. However, in order to alleviate the negative effects that long working hours and care tasks have for many people, especially for the most vulnerable, conciliation measures should be applied so that all citizens have the option of carrying out physical activities for their health.
- Different environments and levels of difficulty since the monotony in activities was found to mainly affect the less motivated population, as this type of activity should generally pose a slight challenge. Therefore: the activities should be segmented according to the subgroups revealed by the study—those who wish to repeat the activity, those who wish to repeat it in different environments, and those who wish to repeat it with a higher level of intensity.
- The different groupings in the form of clusters that emerged since each cluster represents a sporting lifestyle in terms of interest, motivation, type of modality, assessment of the competition, and type of practice.
- Finally, though the mere installation of facilities and/or resources without this training means that many people are not motivated to try out activities due to ignorance, fear of not engaging it in the correct way, etc., the dynamization of activities generates interest in autonomously continuing activities following completion, as well as allowing for lonely users to meet other people with similar interests and to be able to carry out activities in groups in the future. Therefore, the periodic revitalization of facilities and the training of trainers seem to be good ideas for future actions.

All of these aspects have been considered by Zuera Municipality, who have offered new activities such as the Day of Street Sport (an outdoor sports day with sport workshops carried on 24 October 2021).

## Figures and Tables

**Table 1:** Review questionnaire for the experts’ validation.

Item	Answer		Comments
	Yes	No	
1. The questions seem relevant to the subject under study.			
2. The order of the questions seems appropriate to me.			
3. Questions are easily understood.			
4. Answers are mutually exclusive when only one option can be chosen (each answer rules out the others).			
5. I have no doubts about how to answer the questions.			
6. The duration of the questionnaire seems adequate to me.			
7. The format of the questionnaire seems adequate and pleasant to use.			

**Table 2:** Descriptive statistics about the participants.

Participants	Variable	Number
Total number of evaluation questionnaires received		131
Total number of evaluation questionnaires validated		129
		**Percentage** (**%**)
Validated/received questionnaires percentage		98.5
Participants percentage in validated questionnaires based on gender	Men	52.7
	Women	47.3
Participants percentage in validated questionnaires based on age groups	<14 years old	53.5
	14–24 years old	20.2
	25–44 years old	14.7
	45–64 years old	11.6
Participants percentage in validated questionnaires based on residence periods in Zuera	<2 years	2.3
	2–5 years	7.8
	5–10 years	7.8
	>10 years	46.5
	No residence in Zuera	35.7

**Figure A1:**
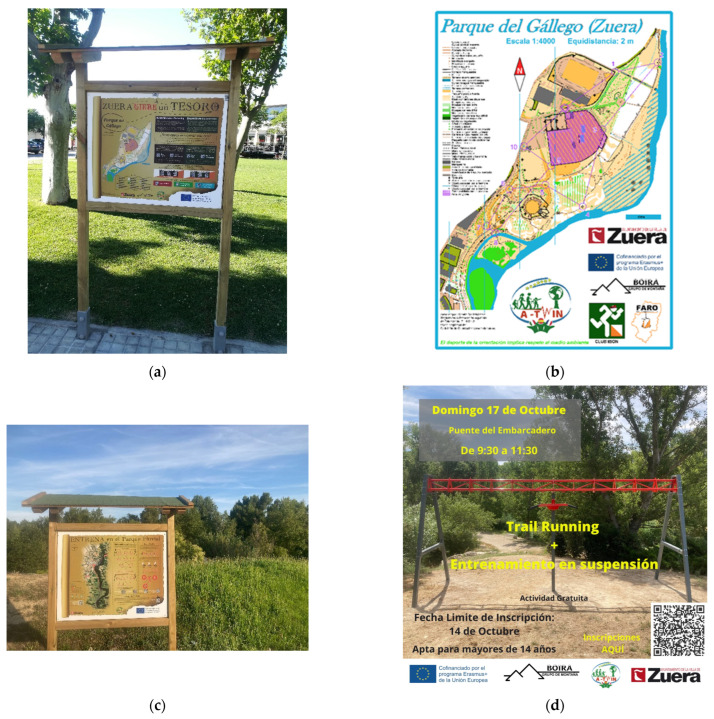
Examples of signposting. (**a**) Signposting of Orienteering Routes “Zuera has a treasure”, (**b**) Orienteering Activity Map “Zuera has a treasure” (**c**) Signposting of Nordic Walking, MTB and TRX training and (**d**) Activity Poster.

## Supplementary Materials

The following supporting information can be downloaded at: https://www.mdpi.com/article/10.3390/ijerph19031161/s1, File S1: Activities’ evaluation sheet.

## Appendix A. Diagnosis Questionnaire

1. Sport facilities and spaces:
  - Does the municipality have routes, signposted routes or heart-healthy circuits?
  - What is the level of difficulty of the routes?
  - What surface area (hectares) do the green areas of the municipality have?
  - Type of the green areas.
  - Does the municipality have outdoor gyms?
  - Does the municipality have outdoor physical activity areas for older adults?
  - Does the municipality have outdoor play areas for children?
  - What other outdoor facilities does the municipality have?
  - Type of outdoor sports activities practiced in these areas.
2. Sport habits:
  - Does the municipality have data on the sports participation and/or sports habits of its citizens? (surveys or statistics of clubs, events, ...).
  - If the council has sport participation data, please indicate the source, if possible the access link, or other reports that can be consulted.
  - If the outdoor sport facilities have a user registration system and/or count of uses, indicate which ones and preferably include the access links.
3. Offered services for outdoors:
  - What clubs or associations of activities in nature does the municipality have?
  - Is there a public record on the sports clubs, associations or entities of the municipality?
  - Type of management.
  - Do municipal services offer a program of activities for outdoor sports?
  - Does your municipality have any app aimed at promoting physical activity?
  - Does your municipality have a sports master/strategic plan?
4. General topics:
  - Are there joint sports activities in the municipality between the city council and educational centers?
  - Are there activities that link sports practice with environmental aspects in the municipality?
  - Are there activities that link sports practice with cultural aspects in the municipality?
5. Suggestions and comments.

## Appendix C. General Descriptive Analysis

**Table A1:** Descriptive statistics of the sample.

Variable	*n* = 393 *n* (%)
x1_Time of residence	
2 or less years	13 (3.31%)
2–5 years	16 (4.07%)
5–10 years	19 (4.83%)
More than 10 years	88 (22.39%)
All my life	257 (65.39%)
x2_Gender	
Men	180 (45.8%)
Women	212 (53.94%)
Other	1 (0.25%)
x3_Age	
14–24	95 (24.17%)
25–44	184 (46.82%)
45–64	114 (29.01%)
Employment situation	
Unemployed	32 (8.14%)
Other	70 (17.81%)
Retired	3 (0.76%)
Employed	280 (71.25%)
Unpaid housework	8 (2.04%)
Family situation	
Other	8 (2.04%)
I live with colleagues or friends	3 (0.76%)
I live with my children or dependents	20 (5.09%)
I live with my parents	122 (31.04%)
I live in couple with children	144 (36.64%)
I live in couple without children	68 (17.3%)
I live by my own	28 (7.12%)
