# Supplementary material for: Physical Activity Participation in Rural Areas: A Case Study
Source: Int J Environ Res Public Health. 2022 Jan 20;19(3):1161. doi: 10.3390/ijerph19031161 (PMC8834896; doi:10.3390/ijerph19031161)
Supplement: Supplementary file 1 [file ijerph-19-01161-s001.zip › ijerph-1510756-supplementary.pdf]

Co-funded by the
Erasmus+ Programme
of the European Union

Dear participant,

We want to express our gratitude for your attendance and effort in the activity performed today. To conclude your participation, you fill out the following survey is needed now that it will help us to be able to evaluate the development of this event. Don't worry, this is totally anonymous and it will take you just two minutes.
THANK YOU VERY MUCH.

AGE: _____ years old **Sex: ☐ Men / ☐ Women**

1. Have you completely finished the activity?

☐ Yes ☐ No

2. How many time are you residing in Zuera?

☐ Less than 2 years ☐ Between 2 and 5 years ☐ Between 5 and 10 years

☐ More than 10 years ☐ I don't reside in Zuera

3. After the experience of this day, do you think this activities programme could be considered as an educational programme?

☐ Yes ☐ No ☐ More information to assess this item is needed

4. How have you participated in this activity?

☐ Alone all the time.

☐ Alone at the beginning, but finally performed with another person.

☐ Alone at the beginning, but finally performed with a group.

☐ In company all the time.

☐ In company at the beginning, but each one has finally done it at his/her own pace.

5. After the activity is finished, and based on your own experience, what would you like in relation to it?

☐ To repeat the activity in the same environment.

☐ To carry out the activity in a similar way but in a different environment.

☐ To carry out the activity in other environments with different characteristics.

☐ To carry out new activitites, taking advantage of the spaces and routes already created.

☐ I would not repeat.

Co-funded by the
Erasmus+ Programme
of the European Union

6. Keeping in mind your effort to carry out this activity, would you change the level?

☐ I would increase the difficulty ☐ I would reduce the difficulty ☐ Difficulty is OK

7. Thinking about activities like the one you have performed today or similar, for each row mark with an 'X' in the corresponding column please, from more negative estimation or fully disagreement (☹️) to more positive estimation or fully agreement (😊):

	☹️	😕	😐	😌	🙂	😊
These activities benefit health.						
These activities keep me fit.						
These activities improve my physical fitness.						
These activities make me feel better.						
These activities relax me.						
These activities make me release stress and tensions.						
These activities are funny.						
Culture can be learned through these activities.						
I think relationship between physical activity and cultural activity is interesting.						
These activities support respect for the environment and knowledge on flora and fauna.						
I think relationship between physical activity and the environment is interesting.						
I would like more activities of this kind were carried out in my town.						

Finally, we request you that all the comments/notes/suggestions/ideas about the activity performed are shown in the following space, so that they help us to improve and be able to detect those aspects we have not initially considered.
